# The Complete Spectrum of Yeast Chromosome Instability Genes Identifies Candidate CIN Cancer Genes and Functional Roles for ASTRA Complex Components

**DOI:** 10.1371/journal.pgen.1002057

**Published:** 2011-04-28

**Authors:** Peter C. Stirling, Michelle S. Bloom, Tejomayee Solanki-Patil, Stephanie Smith, Payal Sipahimalani, Zhijian Li, Megan Kofoed, Shay Ben-Aroya, Kyungjae Myung, Philip Hieter

**Affiliations:** 1 Michael Smith Laboratories, University of British Columbia, Vancouver, Canada; 2 Genome Instability Section, Genetics and Molecular Biology Branch, National Human Genome Research Institute, National Institutes of Health, Bethesda, Maryland, United States of America; 3 Banting and Best Department of Medical Research, Terrence Donnelly Centre for Cellular and Biomolecular Research, University of Toronto, Toronto, Canada; Stanford University School of Medicine, United States of America

## Abstract

Chromosome instability (CIN) is observed in most solid tumors and is linked to somatic mutations in genome integrity maintenance genes. The spectrum of mutations that cause CIN is only partly known and it is not possible to predict *a priori* all pathways whose disruption might lead to CIN. To address this issue, we generated a catalogue of CIN genes and pathways by screening ∼2,000 reduction-of-function alleles for 90% of essential genes in *Saccharomyces cerevisiae*. Integrating this with published CIN phenotypes for other yeast genes generated a systematic CIN gene dataset comprised of 692 genes. Enriched gene ontology terms defined cellular CIN pathways that, together with sequence orthologs, created a list of human CIN candidate genes, which we cross-referenced to published somatic mutation databases revealing hundreds of mutated CIN candidate genes. Characterization of some poorly characterized CIN genes revealed short telomeres in mutants of the ASTRA/TTT components *TTI1* and *ASA1*. High-throughput phenotypic profiling links *ASA1* to TTT (Tel2-Tti1-Tti2) complex function and to TORC1 signaling via Tor1p stability, consistent with the role of TTT in PI3-kinase related kinase biogenesis. The comprehensive CIN gene list presented here in principle comprises all conserved eukaryotic genome integrity pathways. Deriving human CIN candidate genes from the list allows direct cross-referencing with tumor mutational data and thus candidate mutations potentially driving CIN in tumors. Overall, the CIN gene spectrum reveals new chromosome biology and will help us to understand CIN phenotypes in human disease.

## Author Summary

Cancer results from mutations that alter the function of normal genes. The results of these mutations directly lead to the known properties of cancer cells, for example, over-proliferation or resistance to cellular death signals. An additional property of most tumors is chromosome instability (CIN)—the unequal distribution of DNA to the daughter cells following cell division. While CIN appears to be important for the formation of many tumors, the mutations that can lead to CIN are not well understood. Here we used a simple model cell, budding yeast, to systematically identify genes whose mutation can lead to CIN. Our data identify previously uncharacterized CIN genes that we show play a role in the stability of important cellular signaling proteins. Moreover, our results directly predict human CIN candidate genes, hundreds of which are mutated in tumors. Together our data create a resource for understanding new chromosome biology and the genetic basis of CIN in human cancers.

## Introduction

Chromosome instability (CIN), involving the unequal distribution of DNA to daughter cells upon mitosis, is observed in the majority of solid tumors. The precise role of CIN in tumor development is uncertain but it may be an important predisposing factor for oncogenic progression by increasing the likelihood of tumor suppressor loss, gene copy number changes or translocations [1], [2]. Perhaps unsurprisingly, given the shared properties of eukaryotic mitoses, many known CIN genes belong to cellular pathways or structures conserved from yeast to humans (e.g. *BUB1*, *MRE11*, Aurora Kinase) [2], [3]. Mutations that cause CIN may drive tumor formation and progression [2]. Although high-throughput screens for genome integrity are becoming a reality in human cells, the spectrum of human mutations that lead to CIN in tumors is only partially characterized [4]. An ideal role for model organism genetics then would be to identify all cellular processes whose disruption can lead to a CIN phenotype, thus enabling identification and functional studies of candidate genes to focus on particular mutations among those found in a tumor genome.

Most functional genomic screens in yeast have naturally focused on the ∼80% of yeast genes that are non-essential. Indeed, the yeast knockout collection is one of the most valuable genomic resources available. Several collections are now available to assay the functions of essential genes; each allele collection has advantages and disadvantages and only a handful of phenotypic screens have interrogated these collections [5]–[8]. Previous CIN screens of non-essential gene deletions have catalogued the increased frequency of chromosome transmission fidelity (CTF), A-like faker (ALF), Bi-mater (BiM), loss of heterozygosity (LOH), and gross-chromosomal rearrangements (GCR) phenotypes [5], [9]–[13]. All of these phenotypes are considered CIN phenotypes as measured by an increase in the rate of marker loss although the mechanisms predominant in each assay differ. Since non-essential genes have been saturated with genome instability screens, a comprehensive screen of essential genes would create a high quality list of eukaryotic genome integrity pathways.

Here we investigate CIN phenotypes in ∼2000 alleles of 1038 essential genes. When combined with published data for non-essential genes this resource defines yeast genome integrity pathways involving 692 genes and 387 enriched gene ontology (GO) terms. Using sequence orthology and the enriched GO terms to delineate CIN pathways, our data creates a list of cross species candidate human CIN genes. In principle, the yeast CIN gene catalogue described here comprises all conserved eukaryotic genome integrity pathways. Cross-referencing the derived human candidate CIN gene list with somatic mutations in human cancer reveals hundreds of CIN candidates mutated in tumors. Moreover, since tumor genomes typically contain many mutations this reference list of candidate CIN genes could help prioritize functional testing of novel somatic variants.

The CIN gene list also provides biological insights at the level of genome integrity pathways and individual CIN genes. As an example, we conduct a directed secondary screen for telomere length in poorly characterized essential CIN mutant strains. We identify four novel telomere modulators including two subunits of the ASTRA (ASembly of Tel, Rvb and Atm-like kinase) complex, *TTI1* and *ASA1* [14]. ASTRA is an essential seven-subunit protein complex with a proposed role in chromatin biology [14]. Recent work highlights functional interactions among ASTRA subunits in metazoans; namely the TTT complex (Tel2-Tti1-Tti2) and the R2TP (Rvb1/2, Tah1, Pih1) complex which together affect biogenesis of phosphoinositide-3 kinase related kinase (PIKK) complexes [15]–[18]. Therefore, ASTRA likely represents the interaction between yeast TTT, R2TP (or at least Rvb1/2) and a substrate PIKK. Our phenotypic analysis suggests that Asa1p functions with TTT to direct the biogenesis of PIKKs. Genome-wide phenotypic profiling of double mutants by synthetic genetic array (SGA) reveals strong TORC1 defects in TTT-*ASA1* mutants which are likely due to reduced TOR-protein levels. Our data suggest that TTT function is conserved in yeast, and that its uncharacterized interacting partner, Asa1p, functions in the TTT pathway.

## Results/Discussion

### A catalogue of chromosome instability genes

**Figure 1:**
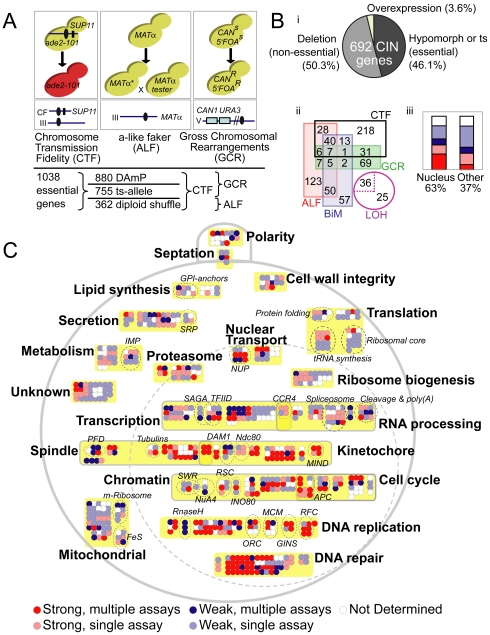
The spectrum of chromosome instability genes in yeast. (A) Schematic of genome integrity screens showing the cellular phenotype measured (top box – i.e. pigmentation for CTF, capacity to mate for ALF, acquired 5′FOA and canavanine resistance for GCR), the chromosomal context of the CIN marker (lower box; blue line indicates chromosome, black oval indicates the centromere, other features are described below) and the type of alleles screened by each assay (bottom). For CTF (left), red pigment accumulates due to a block in adenine production (*ade2-101*) that is relieved in the presence of *SUP11* (black rectangle) carried on a non-essential fragment of chromosome III or VII (CF) (Blue line). Strains carrying the CF will be unpigmented while those that lose the CF will be red. Colonies of mutants with whole chromosome instability will grow with red sectors in an unpigmented colony. For ALF (center), loss of the MATα locus leads to dedifferentiation to an a-mating phenotype. α-mating type mutant strains are mated to a MATα test strain and growth of diploid mated progeny is assessed on selective media, where the ability to mate and form diploids reflects loss, deletion or inactivation of the MATα locus. For GCR (right), *URA3* is integrated near the *CAN1* gene on the left arm of chromosome V where the distal portion of the chromosome arm is non-essential. GCR assay strains are patched onto double counter-selection media where only *ura3*, *can1* strains can survive thus revealing increased rates of terminal chromosome arm deletion and non-reciprocal rearrangements. (B) Overview of CIN gene list. (i) CIN mutations in essential, non-essential and overexpressed genes. (ii) Overlap among five high-throughput CIN phenotypes links 683 of the CIN genes (overlap of 36 LOH mutants (dotted lines) is not shown for clarity). (iii) CIN phenotypic strength in nuclear localized versus other genes. Bars colored according to **1C**. (C) Cellular schematic of 692 CIN genes grouped according to function (Table S1). The large dotted circle represents the nucleus. Colors indicate a strong phenotype (red) or a weak phenotype (blue) where darker represents CIN in multiple assays and lighter represents CIN in a single assay. Yellow boxes and bold labels indicate groups, and dotted circles and italic labels indicate subgroups or protein complexes. Subgroup abbreviations; IMP – inosine monophosphate synthesis; SRP – signal recognition particle; GPI – glycophosphatidylinositol synthesis; NUP – nucleoporins; PFD – prefoldin complex; FeS – Iron Sulfur cluster synthesis; m-ribosome – mitochondrial ribosomal subunit.

To approach a complete list of all CIN genes in yeast we performed overlapping genome instability screens on three collections of essential gene alleles representing 90% of essential genes (Figure 1A). The DAmP (decreased abundance by mRNA perturbation) collection, which disrupts mRNA stability by inserting *KanMX*, encoding G418 resistance, into the 3′UTR of 880 essential genes [6], was screened for CTF [19] and GCR [12]. A collection of 362 ts-alleles created *de novo* using the diploid-shuffle method [5], [10] was screened for CTF and ALF phenotypes and a collection of 755 ts-alleles collected from the yeast community and integrated into a standard genetic background (Z. Li, Charles Boone manuscript in preparation) was screened for CTF and GCR (Figure 1A). The three CIN assays measure different types of genome instability: CTF measures whole chromosome loss, ALF can detect chromosome loss, gene conversion and chromosome rearrangements (i.e. deletions or translocations with DNA loss), and GCR measures chromosome rearrangements, primarily in the form of terminal chromosomal deletions [12], [13]. Overall each allele was screened by two CIN assays, linking 257 essential genes to a CIN phenotype, including a large majority not previously associated with CIN (Table S1). To generate a catalogue of all yeast genes associated with CIN we compiled published genome-wide screens for CTF, ALF, Bi-Mater (BiM), loss of heterozygosity (LOH) and GCR as well as chromosomal marker-loss phenotypes reported in the *Saccharomyces* genome database (SGD: www.yeastgenome.org) for individual CIN gene mutants [5], [9]–[13], [20], [21]. When combined with our data, 692 verified yeast ORFs are mutable to a CIN phenotype (Figure 1B. Table S1). Approximately half of these genes (52%) were either identified by ≥ two independent experiments or showed reproducibly strong CIN phenotypes (Table S1). Proportionally there are more essential CIN genes (323/1156, 28%) than non-essential ones (369/∼4800, 7.7%) although the absolute numbers are similar. In general, CIN genes encode nuclear proteins and nuclear localization correlates with a stronger or higher confidence CIN phenotype (Figure 1B). Based on their descriptions in SGD, published associations, and GO terms, the CIN genes can be grouped into a handful of cellular processes (Figure 1C, Table S1).

The breadth of the CIN gene list suggests that many biological processes protect genome integrity. A large proportion of CIN genes function in predictable pathways (e.g. approximately 40% function in mitosis, DNA replication, repair or modification; Figure 1C). Another 20% of CIN genes function on or near DNA in pathways known to impact genome stability (i.e. transcription, RNA processing, nuclear transport or the proteasome). The remaining 40% of CIN genes work in peripheral biological pathways, (Figure 1C) some of which have established links to genome integrity (e.g. iron-sulfur protein biogenesis) [22] and others with unknown connections to CIN (e.g. tRNA synthesis, GPI-anchors, secretion). The mechanism of CIN for most genes and cellular pathways will require further experiment. However, the entire CIN gene list can also serve as a resource for guiding human CIN candidate gene identification in cancer somatic mutation data.

### CIN-associated cellular pathways and human CIN candidate genes

**Figure 2:**
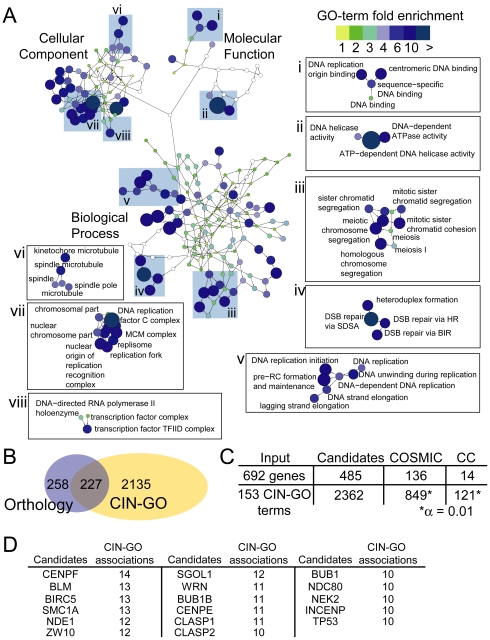
A constellation of GO-terms define CIN pathways and human CIN candidates. (A) Enriched GO terms representing cellular CIN pathways are plotted in the context of the GO-hierarchy. Blue coloration and node size increase with higher fold enrichment. Highlighted sections of the network are marked with roman numerals. (B) CIN gene orthologs and CIN-GO terms generate a partly overlapping candidate gene space. (C) Cross-referencing CIN candidate genes with the COSMIC database and the cancer gene census (CC) databases. Asterisk indicates significantly more mutated CIN-GO genes in COSMIC and CC than the population (Z-test of two proportions).

The comprehensive nature of the CIN screens performed allows a description of the cellular pathways that are most readily mutable to CIN. These pathways may represent those most likely to cause CIN when mutated at random in a neoplasia. We calculated enriched GO terms for the entire CIN gene list using all genes screened as a background gene set (see Materials and Methods). This analysis identified 387 GO terms enriched within the CIN gene list (CIN-GO terms; Figure 2A, Table S2). The enriched terms describe the cellular components (79 terms), biological processes (257 terms) and molecular functions (51 terms) that define CIN phenotypes in yeast. Figure 2A illustrates the network of enriched cellular pathways in the CIN gene list in the context of the GO hierarchy, colored by the calculated fold-enrichment. Highlighted regions of the CIN-associated GO hierarchy illustrate some of the most enriched clusters of terms, especially DNA replication (Figure 2A I, II, V and VII), DNA repair (II, IV), mitotic chromosome segregation (III, VI) and transcription (VIII). Importantly, other less-predictable CIN pathways are also enriched; for example, the mRNA cleavage factor complex (GO:0005849, 4.3 fold), the proteasome (GO:0031597, 4.4 fold), and nuclear import (GO:0051170, 3 fold; Table S2).

As more tumor genomes are sequenced an important task will be associating particular somatic variants, among all the irrelevant, non-functional variants (i.e. passenger mutations) and causative variants (i.e. driver mutations), to particular cancer phenotypes, such as CIN. Since CIN is multigenic (i.e. mutation in one of many genes can lead to CIN) and the complete spectrum of human CIN genes is unknown, the CIN gene list described here could direct the search for CIN-associated variants within a tumor genome. To generate a list of human CIN candidates we compiled sequence orthologs, or in some cases functional orthologs, of the yeast CIN genes, providing a list of 485 human CIN candidates (Table S3). A complementary approach is to define conserved CIN pathways using CIN-associated GO terms (Figure 2, Table S2). This approach has the advantage of capturing genes that belong to known CIN pathways but are not necessarily conserved in yeast. While the CIN-GO terms in principle correspond to eukaryotic CIN pathways, analysis of the terms showed a less specific component that represented vague terms with a low total fold-enrichment. To increase the stringency of this approach we set a cut-off of ≥3-fold enrichment for cross-species comparison. In total 2362 human genes were linked to 153 of the CIN-associated GO terms through 4688 associations (Table S4). The orthology and GO-based human CIN candidate genes represent a partially overlapping sequence space for comparison with somatic mutation data (Figure 2B).

As more somatic variants are identified we anticipate the CIN candidate list serving as a filter to direct phenotypic studies, similar to previous candidate gene based studies (Barber et al., 2008). To assess the present status of this effort we queried the Catalogue of Somatic Mutations in Cancer (COSMIC) and the Cancer Gene Census (CC) with each set of CIN candidate genes [23], [24]. This search identified 136 yeast CIN gene orthologs and 849 CIN-GO based CIN candidate genes with variants in COSMIC and proportionally fewer in the smaller CC dataset (Figure 2C). These mutated cancer genes appear in diverse cellular pathways many of which were not previously associated with CIN. Moreover, the GO-based CIN candidates retrieve significantly more mutated genes than would be expected at random (p<0.01; Figure 2C). Figure 2D shows the CIN candidates associated with 10 or more CIN-GO terms illustrating that this approach yields many known cancer genes (e.g. BLM, WRN, SMC1A, BUB1, TP53) [2], [3].

### Telomere length defects in uncharacterized essential CIN genes

**Figure 3:**
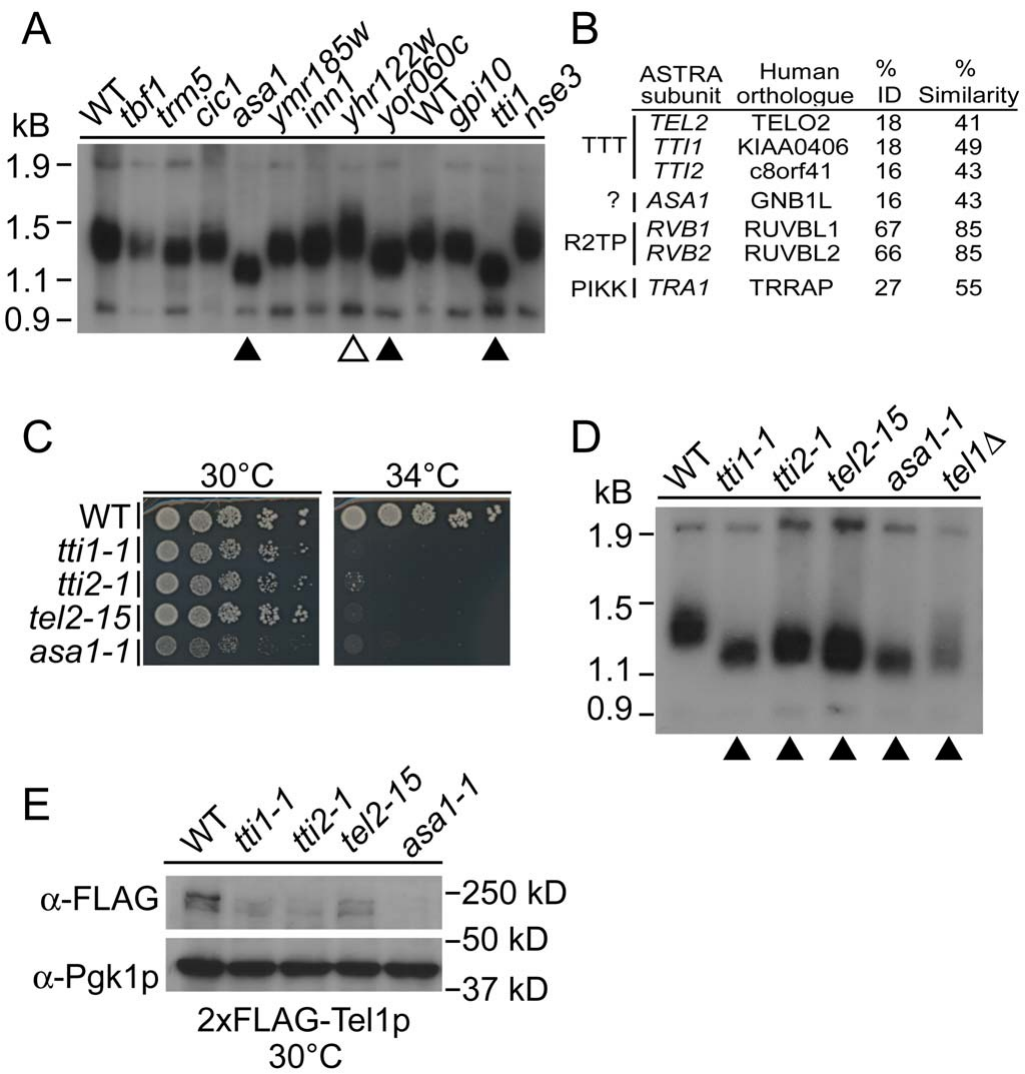
A screen for altered telomeres among poorly characterized essential CIN genes identifies components of ASTRA/TTT. (A) Southern blots of telomere length alterations in indicated mutants. 11 strains are shown, other strains screened had WT telomere length (data not shown). Telomeres are bright bands under 1.5 kb, control bands are seen at the top and bottom of the gel. Triangles indicate short (filled) or long (open) telomeres. (B) Conservation of ASTRA components from yeast to humans. (C) Temperature-sensitive growth of TTT subunit and *ASA1* mutant alleles in spots of 10-fold serially diluted yeast cultures. (D) Southern blot of TTT-Asa1 telomere length as in (A). (E) Western analysis of 2xFLAG-Tel1p in the TTT-Asa1 ts-alleles including α-Pgk1p blots as a loading control.

The CIN gene catalogue identifies a number of uncharacterized or poorly-characterized essential genes (Table S1). Since several of these genes have potential connections to telomeres we assayed telomere length in 20 poorly characterized essential CIN mutant strains. Preliminary southern blots of telomere length showed short telomeres for *tti1-1*, *asa1-1*, and *yor060c-1* and long telomeres for *yhr122w-1* relative to wildtype (WT) (Figure 3A). To confirm these observations we grew these strains at the highest permissive temperature and found reproducibly short or long telomere phenotypes, for the respective strains (Figure S1). While little is known about *TTI1* and *ASA1* and they exhibited only weak ALF phenotypes (i.e. 5- and 3-fold increase over wildtype respectively), we chose to focus on these genes because they are physically associated in *S. cerevisiae* and *S. pombe* in the context of a putative chromatin related complex called ASTRA [14]. In addition, Tti1p forms part of the TTT complex that, along with Tel2p and Tti2p, participates in a PIKK folding pathway which involves the Hsp90 molecular chaperone and the R2TP/prefoldin-like complex [16], [18], [25].

### ASTRA-associated 1 (ASA1) shares telomeric phenotypes with TTT complex mutants

The ASTRA complex contains seven subunits; Tel2p, Tti1p and Tti2p, which comprise the PIKK biogenesis complex TTT; the AAA ATPase complex Rvb1p/Rvb2p; the PIKK Tra1p and Asa1p [14], [16], [18], [25]. Rvb1/2p and Tra1p are highly conserved from yeast to mammals and function in multiple discrete protein complexes [14], [26]. The remaining ASTRA subunits are weakly conserved (Figure 3B) and all but *ASA1* function in PIKK biogenesis [16], [18]. A recent article links the TTT complex, through Tel2p, directly to Rvb1/2 and PIKK biogenesis in the context of the R2TP complex [15]. Therefore, the ASTRA complex may reflect the interaction of TTT, Rvb1/2 and a substrate protein, in this case the PIKK Tra1p [14]. Since the R2TP complex forms a separable functional unit with diverse cellular functions, we chose to focus on the TTT complex and Asa1.

Deletion of *TTI2* has not been reported and we found that, like other TTT subunits, it is an essential gene (Figure S1). Using the diploid-shuffle method we generated *tti2*-ts alleles to complement our set of *TTI1*, *TEL2* and *ASA1* ts-alleles (Figure 3C) [5]. We found that all mutant alleles of TTT components and *ASA1* had telomeres as short as a *tel1*Δ control when passaged at a semi-permissive temperature (Figure 3D). Given the role of the TTT complex in ATM/Tel1p biogenesis we assessed the levels of Tel1p in our mutant strains by western blot (Figure 3E). As expected yeast TTT loss of function mutants lead to Tel1p instability similar to TTT knockdowns in mammalian cells (Figure 3E) [16], [18]. Remarkably, the *asa1-1* allele has a similar reduction in Tel1p levels which is consistent with a common function being executed by the TTT complex and Asa1p. *TEL1* is non-essential but its conserved paralog *MEC1* is essential in yeast carrying a functional *SML1* gene. However, deletion of *SML1* did not ameliorate the ts-phenotype of the TTT/Asa1 mutants consistent with previous observations for *TEL2* mutants (Figure S1) [27]. Overall this data suggests that yeast TTT functions similarly to its mammalian counterpart in PIKK biogenesis and that *ASA1* is a putative functional partner of TTT in PIKK biogenesis.

### High-resolution phenotypic profiling links TTT and ASA1 to TORC1 signaling

**Figure 4:**
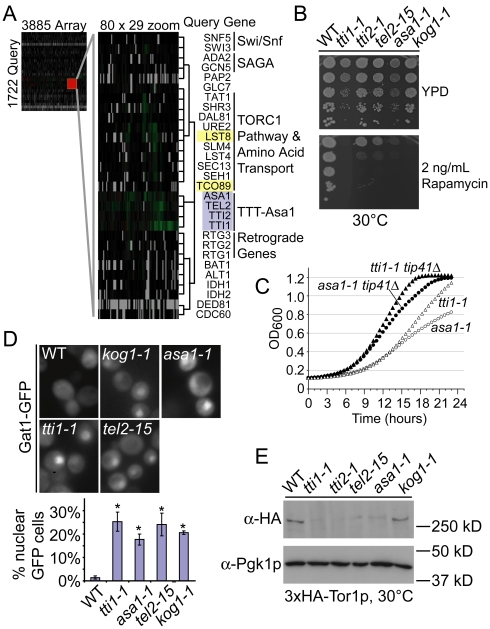
High-resolution phenotypic profiles link TTT and ASA1 to TORC1 function. (A) Hierarchical clustering of genome-wide SGA profiles for TTT and *ASA1* ts-alleles with the global genetic interaction network [29]. Queries are grey and TORC1 components are yellow. (B) Dilution series of TTT-Asa1 ts-allele growth +/− the TORC1 inhibitor rapamycin. (C) Growth curve analysis of hypomorphic *tti1-1* and *asa1-1* alleles in congenic strains +/− *TIP41*. (D) Localization of Gat1-GFP in TTT-Asa1 mutants. Below the sample images a graph shows the mean proportion of cells with nuclear GFP +/− SEM (*  =  p<0.01 compared to WT; *tti2-1* not done). (E) Western blot analysis of 3xHA-Tor1p in the TTT-Asa1 ts-alleles including α-Pgk1p blots as a loading control.

To generate an unbiased phenotypic profile of ASTRA mutants we performed synthetic genetic array (SGA) screens using *tel2-15*, *tti1-1*, *tti2-1* and *asa1-1* as query genes. SGA compares the fitness of arrayed double mutant yeast strains, generated in high-throughput, to the corresponding array single mutants. This technique provides a digital colony size comparison that indicates potential genetic interactions between a query gene (i.e. an ASTRA ts-allele) and the arrayed mutant yeast deletion collection, DAmP allele collection or ts-allele collection [6], [28], [29]. Each screen yielded hundreds of genetic interactions including approximately 100 shared between at least two of the query genes (Table S5). The pattern of genetic interactions with the >5000 array mutants represents a phenotypic profile of each query mutant that indicates functional consequences of the query mutation [28]. We used hierarchical clustering of the four SGA profiles to place them within the context of the global yeast genetic interaction network [29]. The TTT-Asa1 mutant SGA profiles clustered together within the global interaction network (Figure 4A). This co-clustering is not an artifact of our in-house SGA screens because other profiles generated in our lab cluster elsewhere in the network according to their biological functions (data not shown). The SGA profiles support a view of TTT and *ASA1* as a functionally cohesive unit, consistent with the reported physical assembly of these proteins across species [14], [18]. Expanding the TTT-Asa1 cluster to show neighboring genes reveals multiple connections to TORC1 including direct components (e.g. *TCO89*, *LST8*), and downstream effectors (e.g. *DAL81*, *URE2*). In addition, a recent analysis of physical interactions of yeast kinases and phosphatases place TORC1 at the center of a network involving the *RTG1*, *2*, and *3* genes, which control a mitochondria to nucleus signaling cascade called the retrograde response, and components of the chromatin modifying complexes Swi/Snf and SAGA, many of which co-cluster with TTT-Asa1 (Indicated in Figure 4A) [30]. Interestingly, another PIKK, Tra1p, whose human ortholog appears to be affected by TTT, is a component of the SAGA complex, suggesting a possible connection between TTT and Tra1p in yeast [15], [16], [18]. This convergence of physical and genetic evidence strongly supports a role for TTT-Asa1 in the TORC1 pathway.

Like Tel1p and Mec1p, TOR kinases contain the PIKK domain and mTOR is a target of the TTT complex in mammalian cells [15], [18], [25], [31]. Therefore, yeast TTT, and potentially *ASA1*, could directly affect TOR stability or function. Consistently, we found that the TTT-Asa1 mutant alleles were hypersensitive to the TORC1 inhibitor rapamycin (Figure 4B) and arrested in G1 when grown at a non-permissive temperature (Figure S2), similar to alleles of the essential TORC1 component *KOG1* [32]. Additionally, deletion of *TIP41*, a downstream negative regulator of TORC1 signaling, improved the fitness of the sickest representative alleles, *tti1-1* and *asa1-1*, at 30°C (Figure 4C) [33]. Moreover, we observed precocious nuclear localization of Gat1p-GFP, a TORC1 regulated transcription factor, in the TTT, *ASA1* and *KOG1* mutants under nutrient rich conditions, which maintain Gat1p in the cytosol of WT cells (Figure 4D) [34]. Together these data confirm that a defect in TORC1 signaling occurs in TTT and *ASA1* mutants. Importantly, we find that the TTT and *ASA1* ts-alleles, but not WT or *kog1-1* cells, showed decreased levels of Tor1p by western blot (Figure 4E). This data distinguish *ASA1* from other TORC1 pathway effectors (e.g. *KOG1*) and support its function alongside the TTT complex in regulating PIKK stability.

### Conclusions

Here we identified four telomere length regulators including mutant alleles of the yeast TTT complex and an associated factor, Asa1p, whose mutation phenocopies mutation of TTT [14], [16], [18]. Our data link yeast TTT and Asa1p to PIKKs, especially TORC1, consistent with a role in PIKK stability/biogenesis (Figure 3 and Figure 4). The data for yeast TTT are consistent with the current model for mammalian TTT function as a PIKK assembly scaffold [15], while conservation of *ASA1* to mammals is unclear (i.e. the distant ortholog *GNB1L* is not characterized). CIN in the TTT mutants is unlikely to be due to TORC1 defects since other TORC1 components do not appear in the CIN gene list. Instead, the shortened telomeres likely associated with loss of Tel1p, are a probable cause of CIN in the TTT-*ASA1* mutants. Alternatively, another PIKK, such as the DNA damage responsive ATR ortholog, Mec1p, or the histone acetyltransferase component Tra1p could be affected by TTT mutation and lead to genome instability. A complete description of the TTT substrate repertoire, its relationship to CIN, its molecular architecture and its interactions with cellular signals, chaperones and PIKKs remains to be elucidated.

CIN is an important process in oncogenesis and may represent a weakness relative to normal cells that can be exploited for tumor therapy. Indeed, some current anti-tumor therapies act as DNA damaging agents or mitotic spindle inhibitors which could induce a toxic amount of genome instability in the already sensitized tumor cell. Our goal in this study was to create a framework beginning in a simple model organism that facilitates the search for CIN-associated variants in cancer via cross species candidate genes. The CIN gene catalogue creates complementary lists of human CIN candidate genes based on direct orthology and enriched CIN GO terms. In the coming decade, a huge number of tumor genome sequences will be produced via next-generation sequencing. Functional analyses like the one described here can be continually cross-referenced to mutational data to generate candidate genes which are potentially responsible for CIN in tumors. These candidates already hint at functional relevance for numerous observed somatic mutations in cancer (Tables S3 and S4). The task of directly testing the function of human variants is immense and will require a large effort from the community.

## Materials and Methods

See also Text S1.

### Yeast strains and growth

Table S6 is a list of yeast strains and plasmids used. Yeast was grown in rich media at 30°C unless otherwise stated. Plasmid bearing strains were grown in synthetic complete (SC) media lacking the appropriate nutrient. For spot assays, an identical optical density (OD) of cells was serially diluted ten-fold and spotted on the indicated plate at the indicated temperature for 72 hours. Growth curves were performed as described [35]. Briefly, logarithmic phase cultures were diluted to and OD of 0.05 in a 96-well plate in triplicate and grown for 24 hours in a TECAN M200 plate reader at 30°C. Figure 4C shows the middle curve of the three replicates for each strain. None of the TTT-*ASA1* ts-alleles were able to grow at the non-permissive temperature of 37°C, regardless of whether *TIP41* was deleted (unpublished observation).

### Synthetic Genetic Array and CIN screens

SGA was performed using a Singer RoToR essentially as described [28] and was also used to introduce the appropriate chromosomal markers for the CTF (i.e *ade2-101*::NatMX and CFIII or CFVII {*URA3*, *SUP11*}) and GCR (i.e. *pif1*Δ::*HygMX* and *hxt13*::*URA3)* reporter strains [12], [13]. Clustering of genetic interaction profiles was done for genes and arrays by average linkage using Cluster 3.0 [36] and viewed with Java TreeView.

Patches of the *CAN1*, *URA3*, GCR assay strains were replicated to media containing canavanine and 5′FOA at 30°C to screen for GCR exactly as described [12]. CTF screening of ts-alleles was done by streaking CF containing strains onto SC media with 20% the normal amount of adenine exactly as described in [13]. Due to the temperature sensitivity, CTF assay strains were tested in an iterative fashion with respect to temperature. Beginning at 30°C, strains were deemed CTF, wildtype or inviable. Inviable strains were re-tested at 25°C, CTF strains were put aside as putative hits, and wildtype strains were retested at 34°C. The process was repeated with the 34°C strains at 37°C for wildtype strains and 32°C for inviable strains. All the CTF positive strains from any temperature were retested in three independent experiments and a qualitative strength designation assigned as described [13]. Independently generated fragments of chromosome III and VII were tested for each CTF assay strain [13], [19]. Screening of DAmP alleles for CTF was conducted at 30°C. The ALF screen was performed as described in [13] except that the 1 cm^2^ patches of each mutant strain were mated to the MATα test strain at 25°C, 30°C and 34°C to explore a range of semi-permissive temperatures.

### Databases and GO enrichment

Enriched GO terms were calculated at (http://go.princeton.edu/cgi-bin/GOTermFinder) using the hypergeometric distribution to define enriched terms. Terms (downloaded September 28^th^ 2010) with a p<0.05 after Bonferroni correction were considered enriched (Table S2). Human CIN candidate genes were compared to tumor mutations found in the COSMIC database (version 49) [23] and the Cancer Gene Census [25]. The data was obtained from the Wellcome Trust Sanger Institute Cancer Genome Project web site, http://www.sanger.ac.uk/genetics/CGP.

### Telomere length analysis

Telomere length was determined essentially as described [37] except that the probes and ladder were labeled with digoxigenin (DIG) and detected with anti-DIG antibodies according to manufacturers instructions (DIG High Prime DNA Labeling and Detection Starter Kit II; Roche). Mutants for telomere length analysis were chosen primarily based on www.yeastgenome.org descriptions including “Protein of Unknown Function”, and with “putative” or “potential” functions.

### Microscopy

Logarithmic Gat1-GFP expressing cultures were grown in SC media and shifted to 37°C for three hours. Live cells were mounted on concanavalin A coated slides and imaged with the GFP filter set (500 ms exposure) using a Zeiss axioscop and Metamorph software (Molecular Devices) essentially as described [38]. Images were analyzed using Image J (http://rsbweb.nih.gov/ij/index.html). Experiments were repeated in triplicate and the proportion of cells with nuclear staining was counted for at least 100 cells from each experiment.

### Western blotting

Logarithmic cultures at 25°C were shifted to the indicated temperature for 5 hours and harvested by centrifugation (4000 ×g, 2 min). Cell pellets were washed with H_2_O and resuspended in lysis buffer at 4°C for glass bead lysis (50 mM Tris-Cl pH 7.8, 150 mM NaCl, 1.5 mM Mg Acetate, 10% Glycerol, 0.5% Triton X-100, 1 mM DTT, 10 mM Na PPi, 5 mM EDTA, 0.1 mM NaVO_4_, 5 mM NaF, 1× complete protease inhibitor (Roche). Cell lysate was centrifuged (10000 ×g, 5 min) and the supernatant retained. Equal amounts of protein (quantified by Bradford Assay reagent, Bio-Rad) were run on SDS-PAGE gels (10% for Pgk1p, 6% for Tel1p or Tor1p), transferred to nitrocellulose membranes and probed with the indicated antibodies.

## Supporting Information

Figure S1 Confirmation and additional TTT and *ASA1* mutant phenotypes. (A) Confirmation of telomere length phenotypes for the four uncharacterized CIN ts-alleles. Ts-alleles were grown at the indicated temperature on YPD plates through three passages before blotting for telomere length. (B) *TTI2* is an essential gene. The *TTI2* open reading frame (*YJR136c*) was disrupted in diploid yeast with a *URA3* or *KanMX* (not shown) cassette. Shown are the haploid progeny of dissected spores from a *tti2*Δ::*URA3/TTI2* heterozygote. All viable spores were *ura*-, circles denote where the presumptive URA+, *TTI2* knockout spores, were placed. (C) *SML1* deletion has no effect on the temperature sensitivity of *tel2-15*, *tti1-1*, *tti2-1* and *asa1-1* strains. Equal ODs of indicated strains were spotted as ten-fold serial dilutions at the indicated temperatures and visualized after 3 days growth.
(TIF)

Figure S2 G1 cell cycle arrest of TTT and *ASA1* mutant alleles. The DNA content of indicated strains, harvested from logarithmic phase cultures at 25°C or shifted to 37°C for 5 hours, was compared using FACS profiles. TORC1 signaling senses the nutrient status of cells and controls exit from G1 [39]. Therefore the *kog1-1* ts-allele (*KOG1* is an essential TORC1 component) is included as a control.
(TIF)

Table S1 Yeast CIN gene master list including this study and literature.
(XLS)

Table S2 Enriched gene ontology terms associated with complete CIN gene list.
(XLS)

Table S3 Human orthologs of CIN genes and tumor associated mutations.
(XLS)

Table S4 Gene ontology based CIN candidate genes and there mutational spectrum in cancer.
(XLS)

Table S5 SGA results for TTT and *ASA1* ts-alleles.
(XLS)

Table S6 Yeast strains and plasmids used in this study.
(XLS)

Text S1 Expanded description of methods specific to supporting information and to support the main text Materials and Methods.
(DOC)
