# Supplementary figures and images for: The Complete Spectrum of Yeast Chromosome Instability Genes Identifies Candidate CIN Cancer Genes and Functional Roles for ASTRA Complex Components
Source: PLoS Genet. 2011 Apr 28;7(4):e1002057. doi: 10.1371/journal.pgen.1002057 (PMC3084213; doi:10.1371/journal.pgen.1002057)

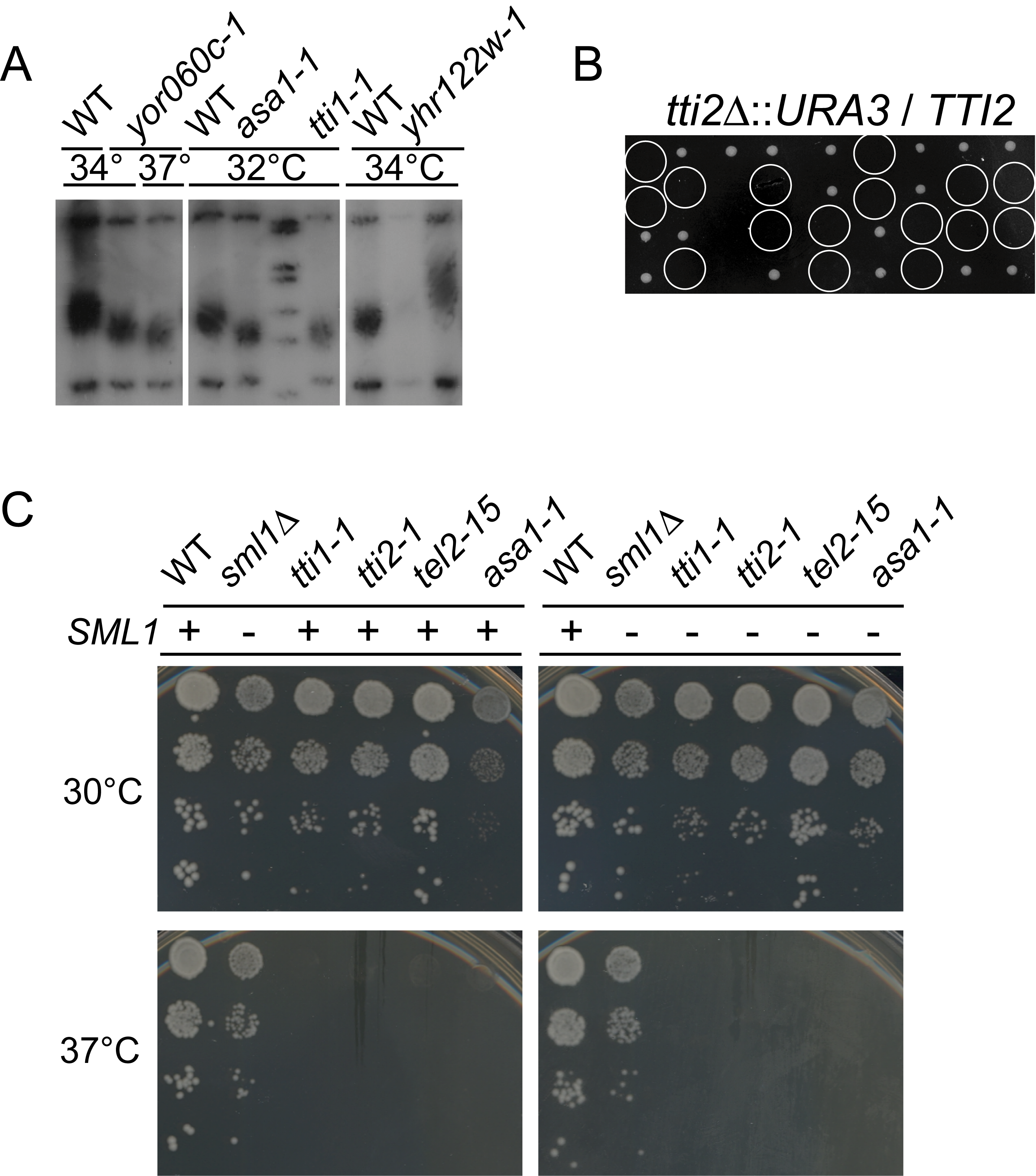

Supplement: Figure S1 — Confirmation and additional TTT and ASA1 mutant phenotypes. (A) Confirmation of telomere length phenotypes for the four uncharacterized CIN ts-alleles. Ts-alleles were grown at the indicated temperature on YPD plates through three passages before blotting for telomere length. (B) TTI2 is an essential gene. The TTI2 open reading frame (YJR136c) was disrupted in diploid yeast with a URA3 or KanMX (not shown) cassette. Shown are the haploid progeny of dissected spores from a tti2Δ::URA3/TTI2 heterozygote. All viable spores were ura-, circles denote where the presumptive URA+, TTI2 knockout spores, were placed. (C) SML1 deletion has no effect on the temperature sensitivity of tel2-15, tti1-1, tti2-1 and asa1-1 strains. Equal ODs of indicated strains were spotted as ten-fold serial dilutions at the indicated temperatures and visualized after 3 days growth. (TIF) [file pgen.1002057.s001.tif]

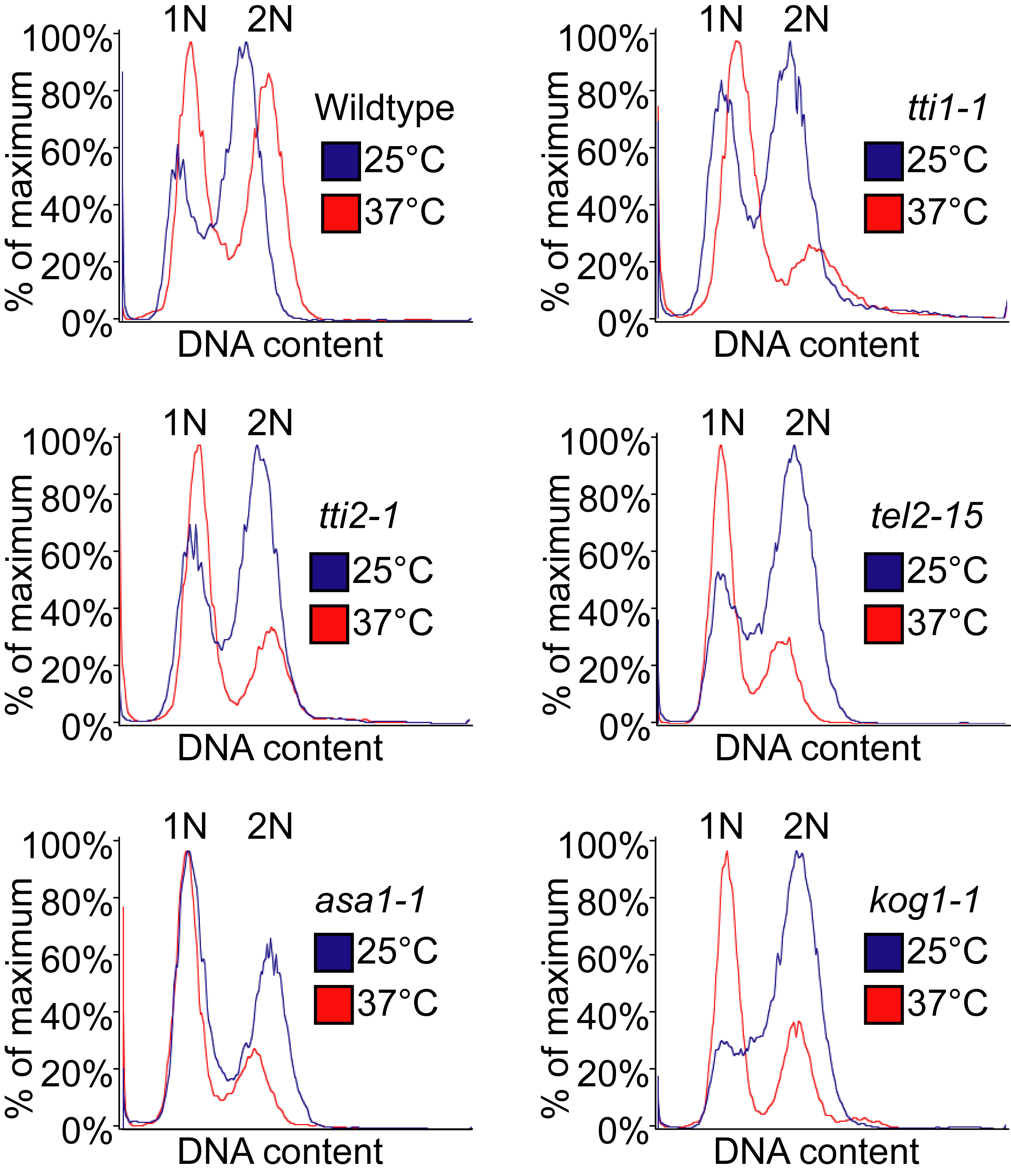

Supplement: Figure S2 — G1 cell cycle arrest of TTT and ASA1 mutant alleles. The DNA content of indicated strains, harvested from logarithmic phase cultures at 25°C or shifted to 37°C for 5 hours, was compared using FACS profiles. TORC1 signaling senses the nutrient status of cells and controls exit from G1 [39]. Therefore the kog1-1 ts-allele (KOG1 is an essential TORC1 component) is included as a control. (TIF) [file pgen.1002057.s002.tif]
