# Supplementary material for: The Complete Spectrum of Yeast Chromosome Instability Genes Identifies Candidate CIN Cancer Genes and Functional Roles for ASTRA Complex Components
Source: PLoS Genet. 2011 Apr 28;7(4):e1002057. doi: 10.1371/journal.pgen.1002057 (PMC3084213; doi:10.1371/journal.pgen.1002057)
Supplement: Text S1 — Expanded description of methods specific to supporting information and to support the main text Materials and Methods. (DOC) [file pgen.1002057.s009.doc]

**Text S1**

**Strain construction**

*TTI2* was deleted using transformation of a *KanMX* or *URA3* cassette flanked with 50 basepairs upstream and downstream of the start and stop codons as described [1]. The *tti2*-ts alleles were constructed exactly as described [2]. Amino acid changes in the TTT-*ASA1* alleles are as follows: *tti1-1* (K41E, S54P, S69N, L272S, N288S, S438P, I636V, L687S, Q758L, L773H, S897F), *tti2-1* (L16P, F104L, K218R, T246M, T297A Q313R), *tel2-15* (A18V, I43V, Y90N, M141T, N178S, S206P, N491S), *asa1-1* (F19S, H75R, T84A, M96T, E101G, K129R, D265G, L323S, N441S).

**Fluorescence activated cell sorting (FACS) analysis**

Yeast cultures were grown at 25°C or 37°C for four hours. An equal optical density (~0.5 OD) of logarithmic culture was washed in 0.2M Tris-Cl pH 7.5 and fixed in 70% ethanol, 0.2M Tris-Cl, pH 7.5 overnight at 4°C. Fixed cells were incubated sequentially with 1 mg/mL RNase A for 1 hour at 37°C, with 1 mg/mL Proteinase K for 2 hours at 50°C, and finally with 3μg/mL propidium iodide, overnight at 4°C. Stained cells were sonicated briefly on low power to separate clumped cells prior to analysis in a BD Biosciences FACS analyzer. The bi-modal distribution of fluorescence intensity corresponding to 1C and 2C DNA content is indicated in **Figure S2**.

**Identification of CIN candidate genes**

Human orthologs of yeast CIN genes were identified first using BLAST searches (e-value< 9^e-06^) and then checked and supplemented using published orthology in the Ensembl and Princeton Protein Orthology (P-POD) databases. Finally, limited manual curation of the list allowed annotation of weaker but valid functional orthologs not picked up in the orthology searches to be included based on supporting literature (**Table S3**).

The CIN GO-term associated human genes were identified by first compiling the enriched GO terms associated with the CIN gene list (**Table S2**). The network shown in Figure 2A was generated using the Cytoscape plugin BinGO [3]. The fold-enrichment (i.e. the ratio of genes per term in the CIN list and genes per term in the background set) was calculated for each term and an arbitrary cut-off of 3-fold enrichment was set to eliminate broad non-specific terms from the analysis (e.g. GO:0044424, intracellular part, 1.25 fold enriched; GO:0050794, regulation of cellular process, 1.79 fold enriched). The list of ≥3-fold enriched terms was queried against the gene ontology via the AMiGO advanced search function and filtered for human genes only (<http://amigo.geneontology.org/cgi-bin/amigo/go.cgi>). Filtering the output of this query to the directly enriched terms only (i.e. removing child terms) generated the list of 2362 human CIN-GO associated candidate genes in **Table S4**. The human CIN-GO term associated genes were downloaded on September 28^th^, 2010.

**SGA scoring and analysis**

The SGA was done according Stoepel et al. (Manuscript in preparation), which is conceptually identical to standard SGA [4]. Briefly, three biological replicates of the mated strains were sporulated and haploid progeny selected using the published canavanine, thialysine, minus histidine selection [4]. For final data collection, single selection plates of the array with G418 (invitrogen) and double mutant selection were expanded in triplicate essentially creating nine replicates (i.e. three biological, three technical) whose colony sizes were captured using a flat bed scanner (Epson). The colony sizes were normalized and converted to pixel area measures using custom software. Statistical analysis of the resultant replicates was performed using R with a script written which compares the nine sets of experimental (i.e. double mutant selection) and control colonies (i.e. single array mutant selection; Stoepel et al., in preparation).

While the scoring system used differs from the large scale SGA score described in [5,6] the range of results produced have similar magnitudes (e.g. approximately -1 to +1) which enabled clustering to proceed without data correction. The whole genome genetic interaction dataset was downloaded from BioGrid and the raw scores extracted. The scores for the interaction of TTT/ASA1 mutant alleles and each array gene were added to this file and subjected to hierarchical clustering using Cluster 3.0. As a control we also clustered in-house SGA data from independent pathways to ensure that clustering occurs according to known biological functions (e.g. the SGA profile of a proteasome subunit clusters with the proteasome, the SGA profile of a TFIID subunit clusters with TFIID; data now shown). In **Figure 4** the green corresponds to negative interactions, red to positive interactions and grey to no data.

**CIN screens and compilation of CIN phenotypic strength**

The ALF screen was conducted on unpublished MATα strains produced alongside the MATa diploid-shuffle ts-allele collection [2]. CTF assays were done with chromosome fragments of ChrIII and VII in parallel and hits were defined as reproducible loss (sectoring) of either chromosome fragment in triplicate (**Table S1**). Putative hits from the ALF primary screen were rested in quadruplicate and those with a >2-fold increase in mating over WT across all four patches are included as hits (**Table S1**). GCR assay strains were constructed in the *pif1*Δ strain background by SGA as described [7]. To partially validate and obtain quantitative GCR data independent of *PIF1*, ten GCR positive DAmP alleles from the primary screen were made in the GCR assay strain RDKY3615 by integrating KanMX immediately after the stop codons (**Table S1**) [7,8]. Fold GCR induction and rate calculations were done as published [7]. Quantitative information was extracted from large scale CIN screens from the literature based on the range of phenotypes reported in each paper and converted into a simple binary phenotypic code (i.e. weak or strong). This was necessary because of the different scales on which the different CIN phenotypes are scored. For CTF assays which range from 1-3 (this study) [2,9,10], a ‘1’ was considered weak and a ‘2’ or ‘3’ were considered strong. For ALF which ranged from 2-80 (this study; [10]), a frequency 2-10 fold over WT was considered weak and ≥10 fold considered strong. For BiM which ranged from 2-6 [2,10,11] ≥4 was considered strong. For LOH which ranged from 2.6 to 272.4 [12], ≥6 was considered strong. Finally for literature GCR phenotypes which ranged from 2-506 [7,13], ≥10 was considered strong.

**Supplemental References**

1. Longtine MS, McKenzie A, Demarini DJ, Shah NG, Wach A, et al. (1998) Additional modules for versatile and economical PCR-based gene deletion and modification in *Saccharomyces cerevisiae*. Yeast 14: 953-961.

2. Ben-Aroya S, Coombes C, Kwok T, O'Donnell KA, Boeke JD, et al. (2008) Toward a comprehensive temperature-sensitive mutant repository of the essential genes of *Saccharomyces cerevisiae*. Mol Cell 30: 248-258.

3. Maere S, Heymans K, Kuiper M (2005) BiNGO: a Cytoscape plugin to assess overrepresentation of gene ontology categories in biological networks. Bioinformatics 21: 3448-3449.

4. Tong AH, Lesage G, Bader GD, Ding H, Xu H, et al. (2004) Global mapping of the yeast genetic interaction network. Science 303: 808-813.

5. Costanzo M, Baryshnikova A, Bellay J, Kim Y, Spear ED, et al. (2010) The genetic

landscape of a cell. Science 327: 425-431.

6. Baryshnikova A, Costanzo M, Kim Y, Ding H, Koh J, et al. (2010) Quantitative analysis of fitness and genetic interactions in yeast on a genome scale. Nat Methods 7: 1017-1024.

7. Smith S, Hwang JY, Banerjee S, Majeed A, Gupta A, et al. (2004) Mutator genes for suppression of gross chromosomal rearrangements identified by a genome-wide screening in *Saccharomyces cerevisiae*. Proc Natl Acad Sci USA 101: 9039-9044.

8. Breslow DK, Cameron DM, Collins SR, Schuldiner M, Stewart-Ornstein J, et al. (2008) A comprehensive strategy enabling high-resolution functional analysis of the yeast genome. Nat Methods 5: 711-718.

9. Ben-Aroya S, Agmon N, Yuen K, Kwok T, McManus K, et al. (2010) Proteasome nuclear activity affects chromosome stability by controlling the turnover of Mms22, a protein important for DNA repair. PLoS Genet 6: e1000852.

10. Yuen KW, Warren CD, Chen O, Kwok T, Hieter P, et al. (2007) Systematic genome instability screens in yeast and their potential relevance to cancer. Proc Natl Acad Sci USA 104: 3925-3930.

11. Daniel JA, Keyes BE, Ng YP, Freeman CO, Burke DJ (2006) Diverse functions of spindle assembly checkpoint genes in *Saccharomyces cerevisiae*. Genetics 172: 53-65.

12. Andersen MP, Nelson ZW, Hetrick ED, Gottschling DE (2008) A genetic screen for increased loss of heterozygosity in *Saccharomyces cerevisiae*. Genetics 179: 1179-1195.

13. Kanellis P, Gagliardi M, Banath JP, Szilard RK, Nakada S, et al. (2007) A screen for suppressors of gross chromosomal rearrangements identifies a conserved role for PLP in preventing DNA lesions. PLoS Genet 3: e134.
